# Fatal Canine Intoxications Linked to the Presence of Saxitoxins in Stranded Marine Organisms Following Winter Storm Activity

**DOI:** 10.3390/toxins10030094

**Published:** 2018-02-26

**Authors:** Andrew D. Turner, Monika Dhanji-Rapkova, Karl Dean, Steven Milligan, Mike Hamilton, Julie Thomas, Chris Poole, Jo Haycock, Jo Spelman-Marriott, Alice Watson, Katherine Hughes, Bridget Marr, Alan Dixon, Lewis Coates

**Affiliations:** 1 Centre for Environment Fisheries and Aquaculture Science (CEFAS), Barrack Road, Weymouth, Dorset DT4 8UB, UK; Monika.dhanjirapkova@cefas.co.uk (M.D.-R.); Karl.dean@cefas.co.uk (K.D.); Steven.Milligan@cefas.co.uk (S.M.); mhamilt8@ford.com (M.H.); Julie.Thomas@gmail.com (J.T.); Chris.j.Poole1961@gmail.com (C.P.); Jo.haycock@hotmail.com (J.H.); Lewis.Coates@cefas.co.uk (L.C.); 2 Taverham Veterinary Hospital, Fir Covert Road, Taverham, Norwich, Norfolk NR8 6HT, UK; Jo.Spelmanmarriott@gmail.com (J.S.-M.); Alice.watson@gmail.com (A.W.); 3 Department of Veterinary Medicine, University of Cambridge, Madingley Road, Cambridge CB3 0ES, UK; kh387@cam.ac.uk; 4 Environment Agency, Dragonfly House, 2 Gilders Way, Norwich, Norfolk NR3 1UB, UK; Bridget.marr@ea.gov.uk; 5 North Norfolk District Council, Holt Road, Cromer, Norfolk, NR27 9EN, UK; Alan.dixon@norfolk.gov.uk

**Keywords:** paralytic shellfish poisoning, starfish, saxitoxins, intoxication, dog, mammals

## Abstract

At the start of 2018, multiple incidents of dog illnesses were reported following consumption of marine species washed up onto the beaches of eastern England after winter storms. Over a two-week period, nine confirmed illnesses including two canine deaths were recorded. Symptoms in the affected dogs included sickness, loss of motor control, and muscle paralysis. Samples of flatfish, starfish, and crab from the beaches in the affected areas were analysed for a suite of naturally occurring marine neurotoxins of dinoflagellate origin. Toxins causing paralytic shellfish poisoning (PSP) were detected and quantified using two independent chemical testing methods in samples of all three marine types, with concentrations over 14,000 µg saxitoxin (STX) eq/kg found in one starfish sample. Further evidence for PSP intoxication of the dogs was obtained with the positive identification of PSP toxins in a vomited crab sample from one deceased dog and in gastrointestinal samples collected post mortem from a second affected dog. Together, this is the first report providing evidence of starfish being implicated in a PSP intoxication case and the first report of PSP in canines.

## 1. Introduction

Paralytic shellfish toxins (PST) are naturally occurring compounds produced by some species of marine phytoplankton, such as *Alexandrium* spp., *Gymnodinium catenatum*, and *Pyrodinium bahamense* [1,2]. These toxins can accumulate in bivalve molluscs through their filter feeding activities, consumption of which by humans and other mammals can result in illness. Consequently, routine regulatory testing of molluscs for the presence of PST is conducted throughout the European Union (EU) as stipulated by EU law [3]. Whilst the occurrence of PST is well recognised and reported in the UK in bivalve molluscs, occurrence in other marine species is less reported. Marine neurotoxins such as PST, domoic acid, causing Amnesic Shellfish Poisoning (ASP), and Tetrodotoxin (TTX) have however been detected in a wide range of marine species including gastropods, tunicates, octopi, cetaceans, crustacea, and fish [4,5,6,7]. Toxin occurrence in fish has been reported in the United Kingdom (UK) previously, including species of flatfish such as Dab, as well as in seals [8]. To date, there are only a few reports of PST in starfish, with all occurrences detected outside of Europe [9,10,11,12]. No testing has been conducted for marine toxins in starfish in Europe to date, presumably given that these animals are considered unsuitable for consumption in European countries.

**Figure 1:**
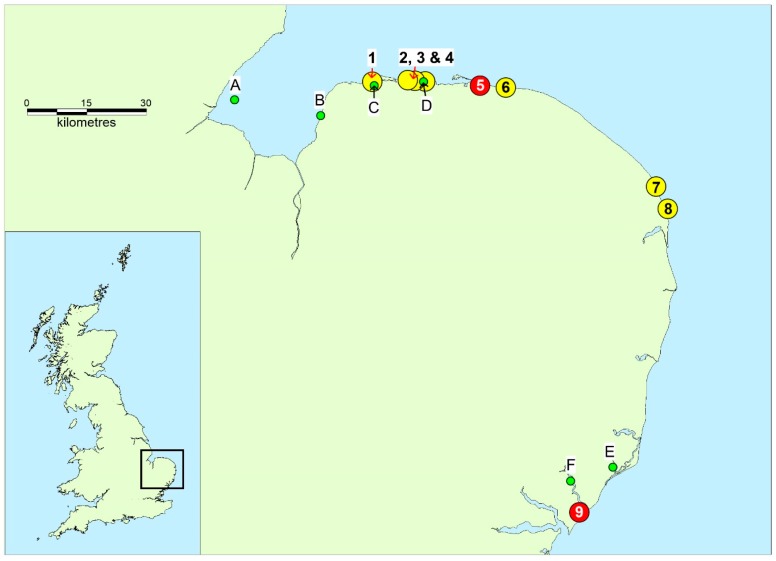
Map of East Anglia showing the locations where dog illnesses and deaths were reported, and nearby shellfish production areas subjected to routine official control testing. Yellow circles indicate dog illness incidents, red circles indicate dog deaths. 1—Burnham Staithe (1 January 2018); 2—Holkham (1 January 2018); 3—Holkham (2 January 2018); 4—Wells-next-the-Sea (31 December 2017); 5—Cley (31 December 2017); 6—Cley (31 December 2017); 7—Horsey (27 December 2017); 8—Hemsby (31 December 2017); 9—Felixstowe Beach (13 January 2018). Green circles denote shellfish production areas: A—The Wash, Toft (mussels); B—The Wash, Stubborn Sand (cockles); C—Brancaster, Loose-J (mussels); D—Blakeney, Wells—The Pool (mussels); E—Butley, Pumping station outfall (Pacific oysters); F—Deben, Stonner Point (mussels).

During late December 2017, gale-force winds affected the east cost of the UK. The prevalent wind was from the NE, gusting to over 30 m per hour, which, together with high spring tides, resulted in large waves hitting the beaches along the county of Norfolk. Towards the end of the month, mass strandings of marine species were reported, including most notably large numbers of the flatfish Dab (*Limanda limanda*) and several species of native scavenging starfish including the common starfish (*Asterias rubens*), the spiny starfish (*Marthasterias glacialis*), and the common sunstar (*Crossaster papposus*). During this time, the minimum and maximum mean daily temperatures in the area were 2.5 °C and 7.5 °C, respectively [13]. The Local Authority and members of the public reported mass fish kills on five different beaches in the area, including Cley and Salthouse (Figure 1). Newspaper reports and social media from around this time described sicknesses in dogs who had visited the beaches along the coast of Norfolk, with most mentioning consumption of beach materials, including fish, prior to illness. In total, eight sicknesses were reported as occurring from Norfolk [14] between 27 December 2017 and 1 January 1 2018 (Figure 1). 

An additional incident (incident 9; Figure 1) was reported as occurring later in January 2018 further down the east coast in the county of Suffolk. Overall, throughout the region, two of the affected dogs were reported as dying within two hours of consuming beach materials. Consequently, the aim of this study was to investigate the potential for the presence of marine neurotoxins in stranded materials. Samples of fish and starfish, together with clinical and pathology samples, were subjected to analysis for toxins to assess any likelihood of marine toxins being a causative factor in the various reports of deaths and illnesses in January 2018.

## 2. Results

### 2.1. Canine Observations

On 31 December 2017, a family with five dogs walked along Cley Beach, at the village of Clay Next Sea, Norfolk, E. England. The family observed the strandline, containing hundreds of dabs, starfish, as well as several dead seal pups. Two of the five dogs were seen to consume material from the strandline. Dog 1, a 28 kg, four-year old Golden Retriever was observed to eat dab as well as a “peach-coloured species”, most likely a starfish or a crab, as testified by the owner. Dog 2, a 12 kg Cocker Spaniel, was seen to eat multiple Dab fish, with no observations of eating starfish made by the owners.

Dog 1 began vomiting on return to the family car, approximately 30 min after the first ingestion. Five to six episodes of yellow-peachy coloured vomit were produced. The dog was not interested in drinking, appeared subdued, but jumped into a car as normal. The dog was subsequently unattended for 10 min followed by a further 40–50 min. At this point, the dog was found in what appeared to be a normal sleeping position, on her right side. On examination, the dog did not respond, and the owners realised she had died. The other dogs in the car were calm. In total, the overall time between ingestion and death was estimated to be 1.5 to 2 h. 

Dog 2 did not appear to exhibit any signs of being unwell until later in the evening of 31 December 2017. She experienced two to three episodes of vomiting but still appeared to be alert and happy. The dog ate and drank water as normal. The following morning, dog 2 had brought up her dinner from the previous night. It was light brown in colour and of a stringy mucus consistency. The other dogs were not interested in eating the regurgitated dinner, which would have been normal behaviour according to the owners. During the morning, the dog was observed to struggle to urinate, possibly a little distressed by her back end. She also appeared roached. On further examination, it was noted that her gums were tacky, and the elasticity of her skin was reduced, inferring dehydration. At this point, the dog was becoming subdued and was taken to the veterinary practice. The remaining three dogs were not seen to consume any substances from the beach and exhibited no signs of illness.

On 13 January 2018, a walker and his seven-year old, 31 kg Siberian Husky dog (Dog 3), walked along the beach at the hamlet of Felixstowe Ferry, Suffolk, E. England, close to the outlet of the Deben estuary. The stony beach was generally devoid of any marine life, although at 10:45 a.m., the dog was observed to eat a shore crab, close to the water’s edge. Because of the position of the crab on the beach and the state of the tide, it was estimated that the crab would have been exposed on the beach for only 15–20 min. Within 30 min of eating the whole crab, the dog was seen to vomit 7–8 times. After lying down for several minutes, the dog attempted unsuccessfully to stand and walk, whereupon he was observed to have paralysis in the hind legs, being able only to drag forward using the front legs. Within the next 10 min, during a car journey to the veterinary practice, the dog lost consciousness. On arrival at the vet, heart massage was performed and oxygen provided, with adrenaline injection to the heart. After 15 min, the dog was pronounced dead. In total, 60–90 min passed between consumption of the crab and the time of death. No further tests were made and no *post mortem* examination was performed.

### 2.2. Veterinary Results and Treatment

Dog 2 presented on 1 January 2018 and was depressed in mentation, showing signs of dehydration and hypovolaemia (loss of fluid from the circulatory system). Blood tests showed that the liver enzymes were normal. She was treated with antibiotics and intravenous fluids and was considered to be more stable after 24 h. After 24 h, blood tests showed elevated liver enzymes. She also had markedly raised inflammatory protein levels. She was started on liver support supplement and stayed in the hospital until 5 January 2018 when blood tests showed that the liver enzyme levels were reducing and she was discharged. The animal did not show any signs of neurological toxicity during hospitalisation.

### 2.3. Canine Post Mortem Assessment

The deceased canine from incident 5 (Dog 1) was referred to the Department of Veterinary Medicine, University of Cambridge, for post mortem examination. Macroscopic post mortem examination revealed dilated cardiomyopathy, with presumptive mild to moderate fibrosis of the ventricular endocardium and myocardium. The relevance of the cardiac pathology to the death of the dog was undetermined. There was no macroscopic or microscopic evidence to support a toxic aetiology for the sudden death of the dog, but it was noted that death was peracute, and so macro- and microscopic morphological lesions associated with an intoxication may not have had time to manifest. There were also extensive changes attributed to post mortem autolysis associated with a post mortem interval of approximately 48 h with storage of the cadaver at >4 °C for the majority of this time.

### 2.4. Toxin Analysis

LC–UV analysis showed no detectable levels of the ASP toxin domoic acid in any of the studied samples. LC–MS/MS confirmed that TTX was also not detected in any of the marine organisms or pathology samples. Consequently, the analysis continued solely with the analysis of samples for PST.

#### 2.4.1. LC-FLD

**Figure 2:**
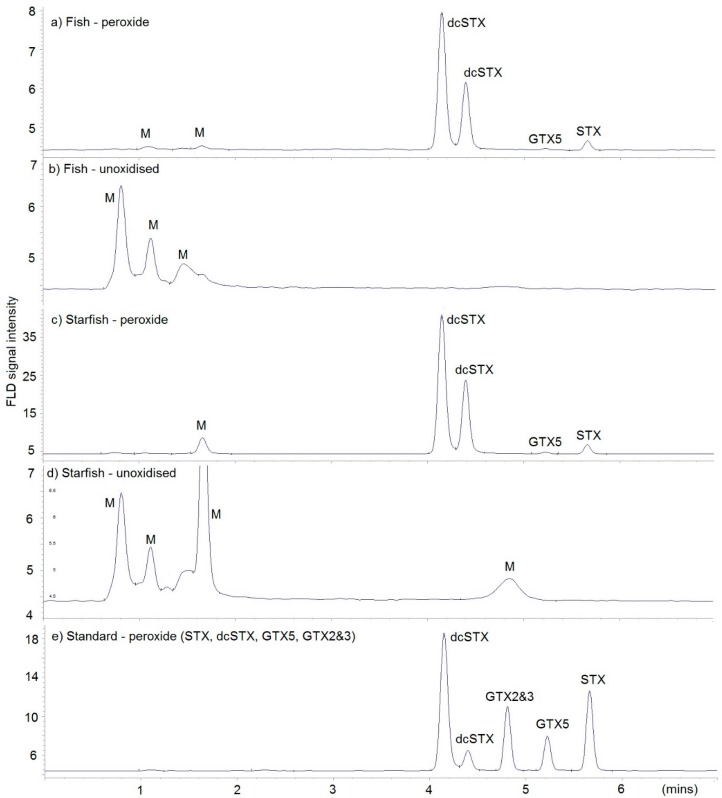
Example LC–FLD chromatograms of (**a**) Dab 1 fish following peroxide oxidation; (**b**) Dab 1 fish—unoxidized extract; (**c**) Starfish 1 following peroxide oxidation; (**d**) Starfish 1—unoxidized; (**e**) toxin standard containing STX, dcSTX, GTX5, and GTX2&3. M = matrix peak.

LC–FLD analysis identified PST in the two dab and starfish samples received from Cley Beach and Felixstowe Ferry Beach, with the confirmation of fluorescent chromatographic peaks present at the same retention time as those generated from calibrants prepared from certified reference materials (CRMs) (Figure 2). A quantitative analysis showed the highest concentrations of PST in the starfish samples, with total concentration of 1516 µg saxitoxin (STX) eq/kg in the Norfolk sample (Starfish 1) and 22,440 µg STX eq/kg in the Suffolk sample (Starfish 2) (Table 1). The results identified three saxitoxin analogues, with decarbamoyl saxitoxin (dcSTX) the most prevalent, followed by STX and gonyautoxins 5 (GTX5), making up approximately 90%, 10%, and <1% of the total toxin load, respectively, in terms of STX equivalents on average in the Norfolk starfish sample. The two fish samples showed PST at approximately 10% of the concentrations measured in the Norfolk starfish collected at the same time. DcSTX and STX were the only toxins detected by LC-FLD, and total PST were 148 and 566 µg STX eq/kg, respectively. The vomit containing partially digested crab from the dog 3 was found to also contain high concentrations of PST, with total PST being 3329 µg STX eq/kg by LC-FLD.

A number of samples of bivalve molluscs were also analysed for PST as part of the official control monitoring programme during December 2017 and January 2018. These were taken from each of the shellfish production areas highlighted in Figure 1. These included mussels, Pacific oysters, and cockles. Without exception, all LC–FLD results showed no detectable levels of PST. Results obtained from the five shore crab samples from Suffolk showed that these samples contained only trace levels of dcSTX, below the method limit of quantitation (LOQ).

#### 2.4.2. LC–MS/MS

**Figure 3:**
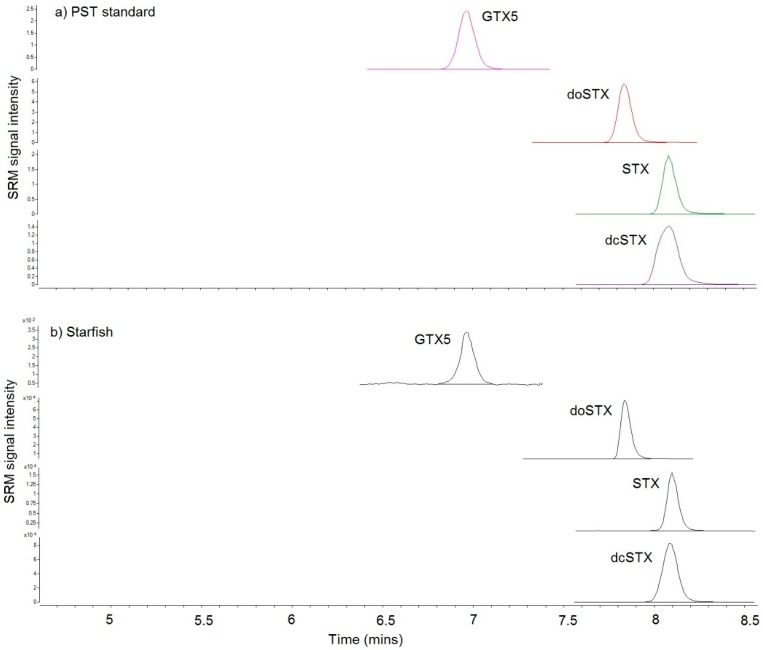
Example of LC–MS/MS Selected Reaction Monitoring (SRM) chromatograms of (**a**) analytical standard; (**b**) starfish sample.

SRM chromatograms obtained following LC–MS/MS of fish and starfish samples confirmed the presence of some PST analogues in all the fish and starfish samples (Figure 3). The total PST concentration was 1083 µg STX eq/kg in the Norfolk starfish, with similar relative proportions of dcSTX, STX, and GTX5 quantified by LC–FLD analysis (Table 1). An additional toxin, deoxydecarbamoyl saxitoxin (doSTX), which cannot currently be analysed by the routine LC–FLD method, was also detected by LC–MS/MS. The concentrations of PST were confirmed as being highest in the Suffolk starfish sample, with a total of 14,439 µg STX eq/kg. The crab sample vomited by dog 3 was also confirmed as containing significant levels of toxins, with a total of 2537 µg STX eq/kg, by the LC–MS/MS method. The toxin profiles averaged 85 ± 10% for dcSTX, 13 ± 10% for STX, and approximately 1% each for GTX5 and doSTX. Whilst the mean proportions of STX were higher on average in the dab samples in comparison to the partially digested crab and starfish, there was no statistical difference calculated between the toxin profiles across the three sample matrices (Anova, single factor, 95% confidence). Out of the five shore crab samples collected from Felixstowe Ferry, only one showed trace concentrations of dcSTX, at 2 µg STX eq/kg, with no other PST detected. 

Overall, for samples containing significant concentrations of PST, LC–MS/MS provided confirmation of the toxins identified by LC–FLD, and the quantitative levels were generally quite similar between the two methods and within the level of variability expected on the basis of the levels of measurement uncertainty associated with both methods. It is also recognised that the differences may also relate to the performance characteristics of the two methods, which remain unvalidated in these species because of the lack of a requirement for routine analysis of such matrices. 

#### 2.4.3. Pathology Samples

The LC–MS/MS analysis revealed the presence of PST in pathology samples taken from dog 1, including the gastric and small intestinal contents, the spleen, liver, and kidney. The highest concentrations per gram of tissue provided were found in the contents of the small intestine, with a total of 164 µg STX eq/kg tissue. In total, 1.25 g of material was sampled from the small intestine. Consequently, the results showed at least 0.2 µg STX equivalents to be present in the contents of the small intestine (Table 2). PST concentration in the contents of the stomach was 89 µg STX eq/kg tissue, approximately half the concentration quantified in the small intestine. The toxin profiles measured in both these samples were dcSTX (61 ± 7%), STX (37 ± 7%), GTX5 (2%), and doSTX (1%), in terms of toxin equivalents. These profiles showed similarities to those determined in the fish and starfish samples, but with a higher relative proportion of STX in the dog tissues. Trace amounts of dcSTX only were detected in the liver, kidney, and spleen of dog 1. No toxins were detected in the brain (formalin fixed tissue) of dog 1 or in the blood sample taken from dog 2 (Table 2).

## 3. Discussion

During early January 2018, in the county of Norfolk, E. England, multiple dog illnesses were reported after visiting beaches, specifically following consumption of dead fish, starfish, and/or crab. The timing of these events coincided with mass strandings of marine species following a severe winter storm activity along the North Sea coast. One dog death was recorded from this area on New Year’s Eve. Two weeks later, a second dog death was reported from Suffolk, a more southerly region of East Anglia, following the consumption of a crab from a beach. To date, there have been no reports of intoxication in British dogs following consumption of seafood or marine species contaminated with saxitoxins [15] and, to our knowledge, there is no published evidence for marine-related canine intoxications. As such, these multiple incidents are highly unusual and, to our knowledge, the first ever reported in the UK. The rapid death of the two dogs from different beaches, together with the observation of paralysis in one of the dogs strongly suggested the potential for a rapid neurotoxic reaction, potentially from marine toxins, as intoxication from PST in mammals is known to begin between 30 min and two hours after the ingestion of contaminated food products [16]. The likelihood of PST being present in marine species along the beach, however, was thought to be very low, given the time of year of this incident. Historically, PST occur in shellfish during the warmer months in the UK, between April and September [17]. Furthermore, there have been no recent reports of PST-producing phytoplankton in the water around the Norfolk coast, and none of the bivalve shellfish collected from the closest classified production areas have shown any detectable levels of toxins in recent years. Nevertheless, given the observed symptoms and subsequent death in two of the dogs, PSP intoxication appeared a possibility.

Results returned by two independent chemical detection methods evidenced quantifiable levels of PST in both dab fish samples and in the starfish samples from both regions. The highest concentrations of toxins were reported in the starfish collected from the Suffolk beach, two days after the second dog death. Worldwide, there are relatively few papers describing PST in starfish species [9,10,11] and no reports of starfish being implicated in PSP intoxication cases [18]. With these studies based on samples from Japan and Taiwan, there is no reported evidence to date of starfish from Europe being affected by PST. The concentrations of total PST quantified in the first starfish sample in this study are similar to those quantified in previous studies from Japan and Chile, where total toxin concentrations ranged from 1440 to 2250 µg STX eq/kg and from 397 to 1353 µg STX eq/kg, respectively. However, the levels quantified in starfish from Suffolk in this study were found to be ten times higher than those previously reported, with a maximum total PST level of 14,439 µg STX eq/kg by LC–MS/MS and 22,400 µg STX eq/kg by LC–FLD. Such concentrations are thought to pose a serious risk to any mammals known to eat such species, which could include either terrestrial or marine mammals [16].

The accumulation of potent neurotoxins such as saxitoxins is well known in bivalve molluscs, given their filter-feeding activity and impact on human food safety. Following the accumulation of toxins in molluscs, however, there are other carnivorous marine species which, through scavenging or predation feeding activities, can become poisonous [4,18]. Other species in the food chain, such as fish, birds, cetaceans, and other marine mammals, can also be affected [8,19,20,21]. 

PST in starfish was considered to originate as a consequence of feeding on detritus [11], whilst [9] reported *A. amurensis* was contaminated directly through feeding on bivalves. In Chile, the PSP toxicity in the starfish (*Stichaster striatus*) was also attributed to the capturing of toxins through feeding on PST-contaminated clams and mussels [12]. [10,22] described the presence of primarily TTXs in *A. scoparius*, together with low concentrations of some PST [10]. The authors further reported the presence of the gastropods *Veremolpa scabra* and *Umborium suturale* inside the stomachs of the starfish, with both species found to exhibit PSP toxicity. As such, there is evidence from the literature for the accumulation of PST and/or TTX in starfish following the consumption of other toxic marine animals such as gastropods and bivalves. In addition, feeding on other neurotoxic benthic marine species, such as flatworms and ribbon worms, can result in the toxification of PST or TTX-bearing organisms such as starfish and fish [23,24,25,26], as well as other marine species such as squid [27] and octopus [28]. Also, there is no published data describing the depuration rates of PST in starfish. Animals might remain toxic for long periods of time, as observed for other marine species such as the Alaskan butter clams [29], with PST becoming chemically bound to tissues and persisting for up to two years after the ingestion of the toxins [30]. The starfish may therefore remain toxic for long periods of time after the original PST food source has detoxified or been removed from the environment. However, experimental laboratory work would be required to determine the rates of depuration to prove such a hypothesis.

Consequently, considering the known feeding habits of starfish on bivalves, there is the potential for the uptake of toxins through feeding processes. Interestingly, however, there are no records of quantifiable concentrations of PST in any bivalve species collected anywhere in East Anglia since 2001. *Alexandrium* species are detected in East Anglia only sporadically and nearly always during the summer periods. Since our laboratory records began in 2005, there has been only one water sample containing PST-producing phytoplankton in the winter, collected in November 2008 from the Wash on the border of Norfolk, containing 80 cells/L. This latest episode infers that there are either PST-contaminated molluscs in the region, although outside the classified shellfish production areas, or other routes of toxin transfer to starfish, fish, and crabs. There may also be the potential for the winter storm activity to release *Alexandrium* cysts, if present in local seafloor sediments, which could enter the benthic food chain, considering the report of a six-fold higher toxicity in cysts than in motile vegetative cells of *A. tamarense* [31]. However, if cysts were indeed present in this region, it is not clear why *Alexandrium* remains only occasionally detected, and there are no findings of PST accumulation in the nearby shellfish production areas. Further work would be required to assess the presence of cysts in the sediments around the East Anglian coast. 

Various published reports describe the presence of PST in marine fish including cod, skipjack, shark, and parrotfish [21,32]. High numbers of mortalities have been previously shown in caged Atlantic salmon as a consequence of dense *Alexandrium* blooms, with toxins detected in phytoplankton, zooplankton, mussels, lobsters, and fish tissues [33]. In Scotland, PST has been detected previously in the flatfish species dab and plaice from the east coast, with total PST concentrations ranging from 758 to 1020 µg STX eq/kg ([8]). As such, the concentrations determined in the dab in this study are similar to those reported previously from the UK. It has been proposed that the transport of PST through the food chain from phytoplankton via zooplankton to higher trophic levels such as fish is an important mechanism [18].

There are also reports of PST toxicity in crustaceans such as crabs, which have been shown to accumulate toxins through feeding on bivalves. Such feeding patterns have been proven in controlled laboratory environments, with reports of PST uptake in the crab (*Ovalipes catharus*) following consumption of the Greenshell mussel (*Perna canaliculus*) [34,35]. In the wild, PST have been found in xanthid crabs, paddle crabs, horseshoe crabs, kelp crabs, shore crabs, blue crabs, as well as edible crabs, lobsters, and shrimps [2,21,36] and references therein], with links to toxicity in prey bivalves and source phytoplankton demonstrated [37]. There are even reports that some crab species, such as the xanthid crab, which can accumulate toxins to extremely high levels up to 160,000 µg STX eq/kg [38], can actively release PST when handled, inferring that the toxins may be involved in a defence mechanism [22]. An enormous variability in toxin concentrations in populations of crabs has also been described [38]. 

The last known PSP intoxication in the UK in human shellfish consumers was recorded in 1968, with high concentrations of PST reaching 50,000 µg STX eq/kg determined in mussels from the north east of England, resulting in the intoxication of 78 people [39,40]. PST accumulation in mussels continued for subsequent years and was found to be associated with dense blooms of *A. tamarense.* During this time, PST were shown to be transferred to both crabs and lobsters, resulting in crustacean toxicity [19]. Around the same time, dying sea birds, dead fish, and sand eels (*Ammodytes* sp.) were found around the Farne Islands, NE England. Investigations revealed the presence of both highly toxic blue mussels and a range of other toxic bivalve molluscs such as clams (*Venus striatula*) and cockles (*Cardium edule*), which were dead. Many hundreds of seabirds were also found dead along the coastline [19,20]. Subsequently, during 1990, PST at levels below the bivalve mollusc regulatory action limit of 800 µg STX eq/kg were detected in edible crabs sampled between NE Scotland and NE England, with low concentrations also detected in one crab sample from Norfolk. PSP toxins were also detected in the digestive glands of lobsters. Given that toxins were not detected in the edible tissues, the UK government lifted the previous warning against the consumption of crabs from the NE coast of the UK [41]. 

The profile of PST quantified in samples from this study was shown to be consistent between different sample types. All samples, including fish, starfish, and partially digested crab, as well as the canine pathology samples, were dominated by dcSTX, followed by STX, with low relative proportions of GTX5 and doSTX. The similarity in profile provided further evidence linking the toxins in the marine animals to the toxins in the affected dogs, including evidence for toxins in both dog vomit and stomach and intestine content. In comparison, the toxin profile identified here differs markedly from that identified previously in the starfish *Asterias amurensis* found in Japan. In that study, the principle toxins were GTX1–4, dcGTX3, and dcSTX, with GTX1 being the most prevalent [9]. A later study reported the findings of a different species of starfish (*A. pectinifera*) from the same locality, this time exhibiting lower toxicity and a toxin profile consisting primarily of STX and neosaxitoxin (NEO) [11]. The similarity in profile between fish, starfish, and crab is interesting, noting the likelihood of differential elimination or species-specific differences in the bioconversion of toxins [32,37,42,43], as well as the potential for metabolism and PST profile changes following ingestion in mammals [44,45]. PST profiles in UK bivalve molluscs have been found to fall into one of four cluster types, with profiles 1–3 consisting of differing relative proportions of STX, GTX1–4, NEO, and C toxins 1 and 2 (C1&2) analogues. The fourth profile was seen only in the surf clams (*Spisula solida*), consisting solely of decarbamoyl congeners such as dcSTX and dcGTX2&3, due to enzymatic toxin conversion [17,46]. Consequently, the profiles recorded in this study represent a markedly different profile, most likely related to a combination of feeding habits and metabolic interconversion of toxin analogues. Further work would be required, incorporating laboratory feeding studies to properly determine whether the profiles reported here result from either an unknown source of toxins or from toxin transformation.

Pathological examination of dog 1 revealed dilated cardiomyopathy, one of the most common heart diseases in dogs. Unfortunately, post mortem autolysis hindered the histological assessment of the myocardium and the determination of the relevance of this lesion to the death of the patient [47]. Whilst there was no macro- or microscopic evidence of a toxic aetiology for the death of the dog, this is consistent with previous reports in humans where only nonspecific findings, such as pulmonary congestion and edema, were described [44]. In those studies, PST were detected in tissue samples, including the liver, kidney, lung, spleen, and heart, the stomach contents, and body fluids. In this study, the analysis of samples collected at the time of post mortem examination revealed PST in the contents of the small intestine and stomach, as well as lower levels in the liver, kidney, and spleen. Consequently, there is strong evidence for dog 1 being affected by PST, following consumption of the crab and/or starfish from the Norfolk beach. Similarly, the identification of PST in the partially digested crab vomited by dog 3 provides proof that the dog ingested an animal containing high concentrations of toxins.

The ultimate cause of the mass strandings of fish and starfish in particular is of interest, but the reasons cannot be elucidated from this study alone. There are anecdotal reports from local people in the East Anglian region of these strandings occurring from time to time. The washing up of large numbers of starfish and razor clams (*Ensis* sp.) is a regular winter event following winter storms. Some reports described starfish as being dislodged from the seabed as a result of mussel fishermen dredging for mussels, although others describe this as an entirely natural occurrence [48,49]. Elsewhere in the world, mass strandings of marine species at the higher trophic level have been attributed directly to the accumulation of PST and other marine neurotoxins [50,51,52,53,54]. There were two notable mass mortalities of the Humbolt squid (*Dosidicus gigas*) in Canada during 2009 following bioaccumulation of PST from sardine and herring [27], although there was no conclusive evidence that PSTs were the definitive cause [55]. Indeed, some authors have concluded that crustaceans and echinoderms may accumulate PST without any significant health effects [21], with some crustaceans able to tolerate extremely high levels of PST [36,56]. Historically, finfish were thought not to be negatively affected by the accumulation of PST, although there are reports of PST-related mortalities in finfish, such as the Atlantic herring (*Clupea harengus harengus*), where pteropods and bivalve molluscs such as clams and mussels were found in the stomach contents of the deceased fish. Laboratory studies involving the feeding of herring with PST-contaminated *A. tamarense* phytoplankton extracts have also been conducted, showing ill effects in the fish, such as irregular swimming, paralysis, and breathing difficulties, with a 79% mortality rate due to asphyxiation [57]. Other authors report that the responses in finfish to PST are highly variable, depending on factors such as fish age, fish feeding patterns and toxicity of the food source, and that the likely cause of toxicity is a direct accumulation of toxins from algae [58,59,60,61,62]. Further examples of this were found in Argentina, following the observed mortality of chub mackerel (*Scomber japonicus*), with evidence of *A. tamarense* cells in the fish stomachs [63].

Most reports of PST accumulation in fish show that PST transfer to humans does not occur if only fish muscle is eaten, as toxins were found primarily in the digestive system of the fish [64,65]. Only low concentrations of toxins have been detected in the edible flesh [66]. Similarly, with crustaceans, reports suggest that the majority of toxins accumulate in the digestive organs and visceral tissue [34,35], although, again, trace amounts of PST have been detected in edible white meat [67]. Consequently, there is likely to be a higher level of risk to humans and mammals, such as canines, who may consume whole animals. Further study is needed to assess the likelihood of toxin accumulation in fishery products subject to human consumption and any subsequent human food safety risks.

Overall, little is known about the source and fate of PST in nontraditional (non-bivalve shellfish) marine vectors. Consequently, intoxications due to the consumption of such species is difficult to predict [18,68]. In this study, however, there is evidence for high concentrations of PST in fish, crab, and starfish. With a positive identification of the same toxin profile in both dog vomit and dog pathology samples, together with relevant clinical observations of the affected canines, this study has produced evidence for the first reported PSP-related canine intoxication. However, there is still a lack of data regarding the initial source of the toxicity, and more work is required to establish the uptake mechanisms into these nontraditional vectors.

## 4. Materials and Methods

### 4.1. Samples

Marine species (Table 3), consisting of two dabs (*L. limanda*) and three starfish (*A. rubens*), were collected from Cley Beach, Norfolk, on 2/1/18. A sample of three starfish (*C. papposus*) and five shore crabs (*Carcinus maenas*) were also received from Felixstowe Beach, Suffolk, collected on 15 January 2018 and 17 January 2018, respectively. A shore crab (*Carcinus maenas*) sample vomited from an affected dog was also collected and provided. One blood sample was received from the surviving sick dog (dog 2) from the Cley Beach incident (incident 5; Figure 1). This was taken more than 48 h after the onset of the clinical signs. At the time of post mortem examination of dog 1 from incident 5, gastric and small intestinal contents, liver, and kidney were collected and stored at −20 °C, pending further analysis. Tissue from the brain was fixed in 10% neutral buffered formalin. The samples were subsequently transported to the Cefas laboratory on ice. Samples of bivalve molluscs were also obtained through the English official control monitoring programme from nearby classified production areas throughout this period (Figure 1) and subjected to analysis. These included common mussels (*Mytilus edulis*), Pacific oysters (*Crassostrea gigas*), and common cockles (*Cerastoderma edule*). All samples of marine organisms were transported under temperature-controlled conditions to the Cefas Weymouth laboratory. The samples were received within one day and, on inspection, found to be in a suitable physical state for chemical analysis. Once opened, the samples were processed immediately.

### 4.2. Reagents and Chemicals

For the analysis of all sample types, instrument solvents, test reagents, and chemicals were either HPLC- or LC–MS-grade, as appropriate to the assay. Certified reference materials (CRM) used for preparing instrumental calibrants were all obtained from the Institute of Biotoxin Metrology, NRCC, Halifax, Canada. 

### 4.3. Sample Extraction

Samples of bivalve molluscs obtained through the official control monitoring programme were shucked and extracted following internal laboratory protocols, prior to analysis for marine toxins. The two dab samples were filleted to remove bones and fins and homogenised separately using both Waring blenders and UltraTurrax homogenisers. The two starfish samples, each consisting of three animals, were each combined into one sample because of their small size and homogenised in a Waring blender. Subsamples (5.0 g each) of the tissue homogenates were extracted in 1% acetic acid following the single-step dispersive extraction protocol of [69], and taken for PST testing. Additional 2.0 g subsamples were subjected to 50% aqueous methanol extractions prior to liquid chromatography with ultraviolet detection (LC–UV) analysis for the Amnesic Shellfish Poison (ASP) domoic acid, following the method of [70]. 

Samples of partially digested crab together with samples of internal organs and blood from canines were treated on a case-by-case basis, depending on the physical nature of the sample matrix. Between one and eight grams of tissue material and/or bodily fluids were homogenised together with an amount of 1% acetic acid corresponding, where possible, to a 1:1 sample to solvent ratio. Where the combined sample/solvent mixture was found to be too viscous to achieve separation through centrifugation, additional volumes of acetic acid were added prior to rehomogenisation, in all cases noting the exact volumes of tissue and solvent taken. Post-centrifugation (4500 rpm for 10 min), the supernatants were decanted and taken for clean-up. Shore crab samples were shucked, and the entire flesh removed and homogenised. Tissue samples (5.0 g each) were extracted with 5 mL of 1% acetic acid as above.

### 4.4. Extract Clean-Up and Analysis

PST analysis was conducted using two independent methods: LC with pre-column oxidation and fluorescence detection (LC–FLD), the current official control monitoring method in the UK for PSP toxins in bivalve molluscs, and Ultra-high-performance liquid chromatography with tandem mass spectrometry (UHPLC–MS/MS, here abbreviated further to LC–MS/MS). For the LC–FLD method, solid phase extraction (SPE) clean-up, ion-exchange fractionation, and pre-analysis oxidation of acidic extracts of the samples were conducted as per [71]. Full quantitation of the samples, following periodate and peroxide oxidation, was performed against external calibrations prepared in solvent. LC–FLD analysis was achieved using an Agilent 1200 LC-FLD system (Manchester, UK), with separation conducted as per [69]. PST analogues incorporated into the method include saxitoxin (STX), neosaxitoxin (NEO), decarbamoyl saxitoxin (dcSTX), decarbamoyl neosaxitoxin (dcNEO), gonyautoxins 1 to 5 (GTX1-5), decarbamoyl gonyautoxins 2 and 3 (dcGTX2&3), and C toxins 1 and 2 (C1&2). 

For LC–MS/MS analysis, acidic extracts of the samples were first subjected to salt removal using carbon SPE cartridges [72] and then diluted and analysed as per [69]. Selected Reaction Monitoring (SRM) was used for acquisition, with primary (quantitative) and secondary (qualitative) SRM transitions utilized, exactly as per [69]. Toxin quantitation was performed using primary SRMs against external calibrations diluted in SPE-cleaned mussel extract. An Agilent (Manchester, UK) 6495B tandem quadrupole mass spectrometer (MS/MS) coupled to an Agilent UHPLC was used for LC–MS/MS analysis, with a Hydrophilic Interaction Liquid Chromatography (HILIC) UHPLC column (1.7 µm, 2.1 × 150 mm Waters Acquity BEH Amide UPLC column in conjunction with a Waters VanGuard BEH Amide guard cartridge, Waters, Manchester, UK). The method was set up to detect and quantify the same PST analogues as for the LC–FLD, together with the additional analogues gonyautoxin 6 (GTX6), deoxydecarbamoyl saxitoxin (doSTX), and C toxins 3 and 4 (C3&4), in addition to Tetrodotoxin (TTX). All other method conditions were as described by [69]. The toxicity equivalence factors (TEFs) used were based on those reported by [16] and modified by [69].

## Figures and Tables

**Table 1:** Summary of individual and total paralytic shellfish toxin (PST) concentrations quantified in dab, starfish, and vomited crab (µg STX eq/kg) by pre-column oxidation LC–FLD and LC–MS/MS.

	LC-MS/MS					LC-FLD				
Toxin	Starfish 1	Dab 1	Dab 2	Starfish 2	Crab	Starfish 1	Dab 1	Dab 2	Starfish 2	Crab
dcSTX	913	115	320	13,730	2363	1361	132	506	21,680	3175
doSTX	37	1.2	3.7	34	13	na	na	na	na	na
GTX5	7	1.6	4.5	96	8.6	6.2	nd	nd	nd	nd
STX	126	46	70	579	153	149	16	60	759	154
Total	1083	164	398	14,439	2537	1516	148	566	22,440	3329

na = no analysis; nd = not detected.

**Table 2:** PST concentrations quantified by LC–MS/MS (µg STX eq/kg of sample received) in pathology samples taken from Dog 1 and in a blood sample from Dog 2.

Sample	GTX5	doSTX	dcSTX	STX	Total
Small intestine contents—dog 1	3.6	1.3	107.3	51.3	164
Spleen—dog 1	nd	nd	33.0	nd	33
Liver—dog 1	nd	nd	15.1	nd	15
Stomach contents—dog 1	1.6	0.4	50.0	37.3	89
Kidney—dog 1	nd	nd	15.5	nd	16
Brain (formalin fixed tissue)—dog 1	nd	nd	nd	nd	nd
Blood—dog 2	nd	nd	nd	nd	nd

nd = not detected.

**Table 3:** Summary of samples received for study.

Sample	Matrix	Date Collected	Location
Starfish 1	Starfish (*A. rubens*)	2/1/18	Cley Beach ^†^
Dab 1	Fish (*L. limanda*)	2/1/18	Cley Beach
Dab 2	Fish (*L. limanda*)	2/1/18	Cley Beach
*Post mortem* samples—dog 1	Organs (various) and gastrointestinal contents	2/1/18	Cley Beach
Blood—dog 2	Blood	1/1/18	Cley Beach
Starfish 2	Starfish (*C. papposus*)	15/1/18	Felixstowe Ferry Beach ^‡^
Crab/vomit—dog 3	Crab—partially digested	13/1/18	Felixstowe Ferry Beach
Shore crabs	Crab (*C. maenas*)	17/1/18	Felixstowe Ferry Beach

^†^ Cley Beach, Norfolk, incident 5 (Figure 1); ^‡^ Felixstowe Ferry Beach, Suffolk, incident 9 (Figure 1).
